# Analyzing EEG patterns in young adults exposed to different acrophobia levels: a VR study

**DOI:** 10.3389/fnhum.2024.1348154

**Published:** 2024-05-06

**Authors:** Samuele Russo, Imad Eddine Tibermacine, Ahmed Tibermacine, Dounia Chebana, Abdelhakim Nahili, Janusz Starczewscki, Christian Napoli

**Affiliations:** ^1^Department of Psychology, Sapienza University of Rome, Rome, Italy; ^2^Department of Computer, Automation and Management Engineering, Sapienza University of Rome, Rome, Italy; ^3^Department of Computer Science, University of Biskra, Biskra, Algeria; ^4^Department of Computational Intelligence, Czestochowa University of Technology, Czestochowa, Poland; ^5^Institute for Systems Analysis and Computer Science, Italian National Research Council, Rome, Italy

**Keywords:** deep learning, machine learning, electroencephalograms, classification, virtual reality, EEG, acrophobia

## Abstract

**Introduction:**

The primary objective of this research is to examine acrophobia, a widely prevalent and highly severe phobia characterized by an overwhelming dread of heights, which has a substantial impact on a significant proportion of individuals worldwide. The objective of our study was to develop a real-time and precise instrument for evaluating levels of acrophobia by utilizing electroencephalogram (EEG) signals.

**Methods:**

EEG data was gathered from a sample of 18 individuals diagnosed with acrophobia. Subsequently, a range of classifiers, namely Support Vector Classifier (SVC), K-nearest Neighbors (KNN), Random Forest (RF), Decision Tree (DT), Adaboost, Linear Discriminant Analysis (LDA), Convolutional Neural Network (CNN), and Artificial Neural Network (ANN), were employed in the analysis. These methodologies encompass both machine learning (ML) and deep learning (DL) techniques.

**Results:**

The Convolutional Neural Network (CNN) and Artificial Neural Network (ANN) models demonstrated notable efficacy. The Convolutional Neural Network (CNN) model demonstrated a training accuracy of 96% and a testing accuracy of 99%, whereas the Artificial Neural Network (ANN) model attained a training accuracy of 96% and a testing accuracy of 97%. The findings of this study highlight the effectiveness of the proposed methodology in accurately categorizing real-time degrees of acrophobia using EEG data. Further investigation using correlation matrices for each level of acrophobia showed substantial EEG frequency band connections. Beta and Gamma mean values correlated strongly, suggesting cognitive arousal and acrophobic involvement could synchronize activity. Beta and Gamma activity correlated strongly with acrophobia, especially at higher levels.

**Discussion:**

The results underscore the promise of this innovative approach as a dependable and sophisticated method for evaluating acrophobia. This methodology has the potential to make a substantial contribution toward the comprehension and assessment of acrophobia, hence facilitating the development of more individualized and efficacious therapeutic interventions.

## 1 Introduction

Anxiety, as a physiological response, can be traced back to the evolutionary imperative for survival (Yuan et al., 2020). The aforementioned primordial alarm system serves to notify individuals of potential risks, hence promoting a state of vigilance and increased attentiveness toward one's surroundings (Giri et al., 2016). Nevertheless, a significant distinction exists between typical anxiety reactions and clinical anxiety disorders. Regrettably, the latter category, encompassing a range of diseases distinguished by intense fear or apprehension, exhibits a notable prevalence (Juul et al., 2019). Remarkably, around 30% of persons worldwide have these diseases at some stage in their lifetimes (Bălan et al., 2020; Fiani et al., 2023). What is additionally concerning is the influence exerted by these illnesses. These intangible restraints serve as impediments to daily activities, impeding productivity and negatively impacting both professional output and social interactions (Babaev et al., 2018; De Magistris et al., 2022). Individuals afflicted with these conditions frequently experience a sense of entrapment, as they actively avoid situations and stimuli that have the potential to exacerbate their symptoms (Dat et al., 2021; Brandizzi et al., 2022). However, a positive aspect can be identified (Marshall and Bewley, 2015). The medical and psychological fields have made significant advancements in the development of various treatments, therapies, and interventions that provide individuals with comfort, enabling them to regain control over their life (Ponzi et al., 2021; Zhang Y. et al., 2021).

When examining the various subcategories of anxiety disorders, it becomes evident that phobias emerge as a prominent classification (Anderson and Shivakumar, 2013; Iacobelli et al., 2023). Phobias encompass more than mere apprehensions, as they entail heightened and frequently illogical anxieties toward particular circumstances, entities, or organisms (Wiens et al., 2022). The phenomenon of phobia encompasses more than just psychological suffering. Individuals afflicted with phobias may exhibit physiological manifestations that encompass respiratory abnormalities, heightened heart rate, and in severe instances, loss of consciousness (Pepe et al., 2022). It is vital to distinguish between phobias and fears. While fear can be considered a logical reaction to an actual danger, phobias are distinguished by an excessive and illogical fear (Bhola and Malhotra, 2014).

When classifying phobias, they can be divided into three main categories: social phobia, agoraphobia, and specialized phobias (Horváthová and Siládi, 2016). Social phobias are characterized by an incapacitating apprehension of being evaluated by others in social situations. These individuals are frequently characterized as introverted, and their history may be marked by negative social experiences (Baek, 2014). Agoraphobia is a comprehensive word that encompasses apprehensions related to both enclosed locations, such as elevators, and open, densely populated environments (Asmundson et al., 2014). In instances of extreme symptoms, individuals may choose to isolate themselves within their residences due to an overwhelming fear of the external environment (Asmundson et al., 2014). Specific phobias, as their name implies, are anxiety disorders characterized by intense and irrational anxieties associated with specific stimuli. Instances are arachnophobia, which pertains to an irrational fear of spiders (Ganatra and Mistry, 2014), as well as acrophobia, which involves an excessive fear of heights (Huppert et al., 2020), among numerous others. The cognitive contemplation or sheer existence of these stimuli has the potential to immobilize the individual with a profound sense of dread (Kristjánsson et al., 2021).

Exposure therapy has emerged as a viable intervention in the battle against phobias (Chalfant and Bernd, 2014). The objective of this treatment strategy is to facilitate desensitization in individuals with anxieties by systematically exposing them to fear-inducing stimuli in controlled settings, ultimately disrupting their pattern of avoidance (Clemente et al., 2014). According to the American Psychological Association, exposure therapy can be categorized into:

- *In vivo* exposure: the concept of *in vivo* exposure involves the deliberate and systematic immersion of individuals into real-life events that elicit fear or anxiety (Alsaffar, 2021). For individuals who experience arachnophobia, this could entail a structured and supervised exposure to spiders, first with the act of seeing them from a safe distance and progressing toward the eventual act of tactile contact (Alsaffar, 2021).
- Imaginal exposure: imaginal exposure, conducted *in vitro*, involves instructing patients to engage in mental visualization of their phobic stimuli. The efficacy of the intervention is somewhat reduced for individuals with a less pronounced capacity for imaginative thinking (Martial et al., 2018).
- Systematic desensitization: systematic desensitization is a therapeutic therapy that employs a dual strategy, wherein relaxation techniques are initially employed to induce a state of calmness in the patient. Following this, individuals are systematically exposed to stimuli that elicit fear, to diminish the fear response (Oing and Prescott, 2018).
- Flooding: flooding involves a kind of therapy that entails subjecting individuals to direct and strong exposure to the source of their anxiety (Markowitz and Fanselow, 2020). This approach is based on the belief that the overpowering experience of terror will not endure indefinitely.
- Virtual reality exposure (VRE): Virtual Reality Exposure (VRE) is a technique that utilizes contemporary technology to create simulated environments that replicate scenarios that are difficult or dangerous to encounter in real life (Wiens et al., 2022).

Phobias and anxiety disorders, however formidable, can be overcome. Through appropriate intervention and support, individuals have the ability to effectively navigate their anxieties, progressively reducing their influence and ultimately achieving a life that is not controlled by them.

Fear reduction has advanced using virtual reality (VR) and its electrophysiological correlates in exposure therapy, particularly for height and spider phobias. These treatment procedures are evaluated using physiological indicators and machine learning (Bousouar et al., 2023, 2024).

The study by Zhang X. et al. (2021) examines the impact of *in-vivo* and VR exposure therapy on spider phobia in a sample of 100 individuals. Both spider and neutral photos caused significant late positive potential (LPP) and early posterior negativity (EPN) in the electroencephalogram (EEG). All individuals' spider-phobias decreased after therapy. Based on Bayesian analysis, *in vivo* and VR exposure therapy have similar effects, as evidenced by consistent EPN and LPP levels (Zhang X. et al., 2021).

Bălan et al. (2020) introduces a novel real-time VR game methodology for acrophobia. Using deep neural networks (Nail et al., 2024), this new technique automated exposure intensity adjustment. This method, using EEG, HR, and GSR, achieved 73% accuracy in fear detection on a two-choice scale. Another study suggested treating acrophobia with VR. It measures fear using machine and deep learning classifiers. Research suggests that integrating beta rhythm EEG values with GSR and HR can effectively classify terror levels (Bălan et al., 2020; Tibermacine et al., 2023).

In acrophobia treatment, acceptance and commitment therapy (ACT) and virtual reality (VR) were examined. Structured six-session programmes taught patients acceptance, anchoring, and diffusion. Treatment findings showed a significant decrease in avoidance behaviors and an increase in value-based activities, proving the effectiveness of an integrated approach (Çelik et al., 2020).

Electroencephalograms have helped us understand acrophobia in virtual reality. A canyon-themed VR environment was shown to eight experimental participants. The Random Forest classifier is highly proficient in the frontal region, with an accuracy rate of 68.2%. Gamma and high-beta frequency bands were shown to be crucial, with accuracy rates of 61.3 and 57.9%, respectively (Apicella et al., 2023).

In addition, Freeman et al. (2018) used VR to treat acrophobia. A support vector machine was used to distinguish relaxed and worried states with an 88% success rate. The classification was based on ECG, GSR, and respiration data (Freeman et al., 2018). Diemer et al. (2016) conducted a study with 80 participants, half of whom were diagnosed with acrophobia, adding to the current knowledge. Both groups showed physiological anxiety reactions to increasing virtual heights, as measured by GSR and HRV (Diemer et al., 2016).

Machine and deep learning approaches, together with VR technology, may be useful in phobia treatment. Electrophysiological markers for real-time feedback improve therapy knowledge and manage the therapeutic setting. Our comprehensive study examined acrophobia through (1) spectral analysis of brain waves, (2) brain region correlations, and (3) the identification and selection of key features that best reflect the brain's response to acrophobic stimuli. The chosen features were validated using advanced neural network models, such as Convolutional Neural Networks (CNN) and Artificial Neural Networks (ANN), which performed better at classifying EEG signals.

## 2 Methodology and implementation

### 2.1 Dataset

The EEG dataset was carefully compiled from a group of 18 participants who were enrolled at a university (see Table 1), resulting in a consistent profile of young adults with an average age of 25.77 years, standard deviation of 3.44 and age range from 20 to 35 years old. The majority of the student cohort is composed of individuals who have academic backgrounds in technical disciplines. This includes Master's and PhD candidates who specialize in Artificial Intelligence (AI), Mechanical Engineering (ME), and Electrical Engineering (EE). Additionally, there are Bachelor's students who are pursuing degrees in Computer Science (CS) and English Literature (EL). Furthermore, it is worth noting that the cohort also includes Master's students who have chosen to specialize in the fields of Electrical Engineering (EE) and Computer Science (CS). The participants of the study were selected from the University of Biskra, Algeria, using a casual yet purposive sampling approach that involved utilizing personal networks of friends and acquaintances. The research entailed the exposure of participants to four separate visual stimuli that represented different degrees of acrophobia within a Virtual Reality setting. The stimuli utilized in this study were acquired from a specialized company known as PsyTech VR. Furthermore, the researchers obtained electroencephalogram (EEG) recordings during periods of rest from the participants, which served as a baseline for subsequent study. One of the primary focal points of our research entails the meticulous safeguarding of the confidentiality of the participants' data. Prior to the initiation of the electroencephalogram (EEG) recording, all participants were mandated to provide their informed consent by affixing their signatures to their student identification cards, therefore clearly authorizing the utilization of their EEG data for research purposes. The research technique was granted authorization by the laboratory located at the University of Biskra, which is situated in Algeria. The study's credibility and security were enhanced by the inclusion of a medical student with a specialization in neurology and a senior medical student in their seventh year, both of whom were members of the oversight committee.

**Table 1:** EEG responses to visual stimuli for university students.

**Subject No**.	**Gender**	**Age**	**Education field**	**No. of groups**	**Recording time**	**MMSE**
S01	M	34	PhD in AI	4	480 s	30
S02	M	29	PhD in AI	4	484 s	28
S03	M	30	PhD in ME	4	479 s	29
S04	M	29	PhD in ME	4	501 s	28
S05	M	28	PhD in EE	4	483 s	30
S06	M	26	MSc in CS	4	476 s	29
S07	F	26	MSc in CS	4	469 s	30
S08	F	25	MSc in CS	4	482 s	30
S09	M	25	MSc in CS	4	495 s	30
S10	M	25	MSc in CS	4	478 s	29
S11	M	25	MSc in CS	4	482 s	28
S12	M	25	MSc in CS	4	493 s	28
S13	M	24	MSc in CS	4	490 s	29
S14	M	26	MSc in EE	4	505 s	30
S15	M	25	MSc in EE	4	483 s	28
S16	M	20	BSc in CS	4	476 s	30
S17	M	22	BSc in EL	4	501 s	28
S18	F	20	BSc in EL	4	482 s	29

The implementation of tight exclusion criteria aimed to verify that all subjects had no prior medical history of epilepsy or any occurrences of epileptic episodes. All of the participants in the study did not have any previous records of traumatic brain injuries, neurosurgical procedures, or illicit substance use that could be linked to their psychiatric condition. None of the participants included in the study had any recorded medical history of psychiatric problems, alcohol or substance misuse, acute cerebrovascular diseases, or serious infections that impacted the central nervous system. Moreover, it is crucial to recognize that all participants in the study had no previous history of psychoactive substance use, showed no signs of auditory abnormalities, and did not self-report as individuals with Synesthesia. The primary aim of our investigation was to establish a thorough comprehension of the cognitive condition and neurophysiological reactions exhibited by the participants upon exposure to auditory stimuli. The achievement was attained by integrating the findings derived from the administration of the Mini-Mental State Examination with electroencephalogram (EEG) data. The implementation of this enhanced methodology ensured that the recorded electroencephalogram (EEG) responses faithfully and efficiently reflected a representative subgroup of young persons with unimpaired cognitive capabilities.

**Figure 1:**
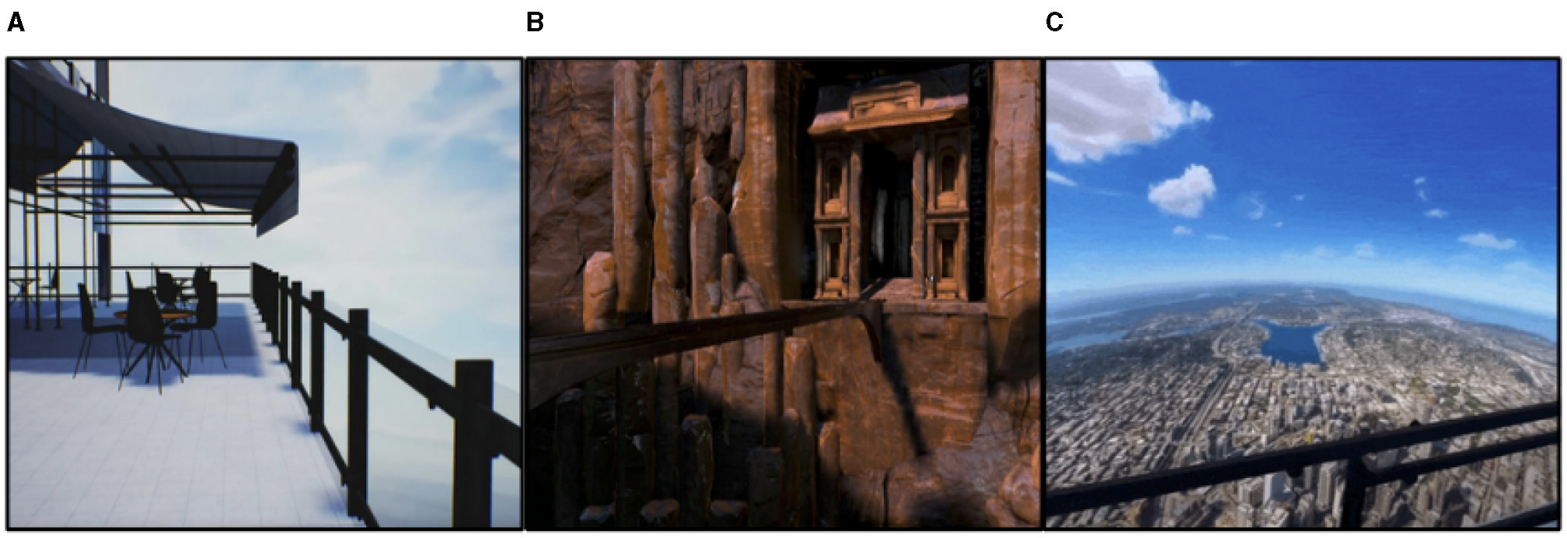
Some of the used VR in this study. **(A)** An apartment balcony, **(B)** a bridge spanning across a canyon, **(C)** a city perspective from a skyscraper.

The data collecting process was carried out via the Emotiv Epoc+ 14-channel EEG headset, which facilitated the comprehensive capture of brainwave patterns in significant cerebral areas. As a result, the dataset has been divided into four distinct categories derived from various virtual reality (VR) settings. These scenes include an apartment balcony, escalators within a retail mall, a restaurant situated in a skyscraper offering a city perspective, and a bridge spanning across a canyon (see Figure 1).

Based on the effect sizes observed in Table 2 and considering the number of subjects in each study, our sample size of 18 subjects appears to be sufficient for the current study. While Apicella et al. (2023) and Tychkov et al. (2023) analyzed smaller samples of 8 and 12 subjects, respectively, Bălan et al. (2020) and Balan et al. (2019) employed similar or smaller sample sizes of 8 and 4 subjects, respectively. Despite variations in sample sizes across studies, the effect sizes reported provide valuable context for our own research. With a sample size of 18 subjects, we aim to leverage these insights to detect meaningful effects within our study population. Moreover, by aligning our sample size with those of previous investigations, we enhance the comparability of our findings and contribute to the cumulative knowledge in the field.

**Table 2:** Effect size.

**References**	**Number of subjects**
Apicella et al. (2023)	8
Tychkov et al. (2023)	12
Bălan et al. (2020)	8
Balan et al. (2019)	4

### 2.2 Preprocessing and feature extraction methodology

After acquiring and preparing the raw data, the feature extraction process involved employing the Short-Time Fourier Transform (STFT) method. The aforementioned methodology is well-suited for the examination of electroencephalogram (EEG) signals in the time-frequency domain.

#### 2.2.1 Preprocessing

The first step in the preparation phase involves the extraction of electroencephalogram (EEG) recordings from the participants. These recordings are typically stored in Comma-Separated Values (CSV) file format. The recordings consist of 14 channels that conform to the MNE standard. The provided format adheres to a universally accepted nomenclature for EEG channels, which is derived from the well recognized 10–20 system for electrode placement with a sampling rate of 128 HZ. The data is then imported into an MNE RawArray object, which offers comprehensive preprocessing and analysis capabilities for neurophysiological data.

In the preprocessing pipeline, two key techniques are employed to enhance the quality of electroencephalogram (EEG) recordings: filtering and Independent Component Analysis (ICA). Initially, bandpass filtering is applied to the EEG signals to attenuate frequencies outside the desired range, effectively removing noise and artifacts. Specifically, a bandpass filter with cutoff frequencies set at 1 Hz (low) and 50 Hz (high) is utilized to isolate relevant neural oscillations while suppressing unwanted components such as baseline drift and high-frequency noise. Subsequently, a notch filter is employed to target the removal of specific frequencies known to be sources of interference, notably the 50 Hz powerline noise. This filter further refines the EEG signals, ensuring a cleaner dataset for subsequent analysis. Following the application of these conventional filtering techniques, Independent Component Analysis (ICA) is employed as a sophisticated method for artifact removal. By decomposing the EEG data into statistically independent components, ICA enables the separation of signal sources, including brain activity and various types of noise and artifacts. Through the identification and removal of artifact-related components, ICA enhances the interpretability and reliability of the EEG recordings, allowing for more accurate analyses of neural dynamics and cognitive processes. In combination, these filtering and ICA techniques play a pivotal role in preprocessing EEG data, facilitating the extraction of meaningful insights from complex neurophysiological signals.

#### 2.2.2 Feature extraction

The Short-Time Fourier Transform (STFT) is a commonly used signal processing technique that is performed to analyse the time-varying frequency characteristics of a signal. The procedure involves dividing the signal into distinct windows of data, where each window consists of 128 samples. Following this, the Fourier Transform is implemented on each individual window, and the magnitude is calculated using the “stft” function provided by the scipy.signal package. The technique described above was executed individually for each EEG channel, resulting in a three-dimensional array consisting of complex values representing time, frequency, and channel, respectively. The mean magnitudes of frequency segments at each temporal instance were determined by calculating the average over specific time intervals. The process of averaging was utilized to reduce the dimensionality of the data and generate a more thorough representation of the frequency content for each channel across the whole recording period. The resulting matrix of features serves as a great dataset for subsequent inquiry or machine learning endeavors. The technique described above is executed in an iterative manner for the electroencephalogram (EEG) data of each individual participant, leading to the generation of a set of feature sets. The sets are then vertically merged to create a comprehensive matrix that encompasses data from all individuals. The size of the extracted features vector is 42, which corresponds to the extraction of three waves from each of the 14 channels.

### 2.3 Classification

The classification of EEG data has gained the attention of many researchers in the last few decades. Various machine and deep learning classifiers, including Support Vector Classifier (SVC), Random Forest (RF), Decision Tree, Ada Boost, Linear Discriminant Analysis (LDA), K-Nearest Neighbors (KNN), and Artificial Neuron Networks (ANN) classifiers, in addition to Multi-Layer Perceptron (MLP) and Convolutional Neural Networks (CNN), have been suggested to generate conclusions about the current state of the subject based on the EEG feature vectors generated during the feature extraction phase.

In our research on the classification of EEG signals collected from individuals experiencing various levels of acrophobia in virtual reality (VR), we chose to use both Convolutional Neural Networks (CNNs) and Artificial Neural Networks (ANNs) as our primary classifiers after careful consideration of the distinct strengths each architecture provides. First, CNNs were chosen because of their inherent capacity to properly capture spatial dependencies within multidimensional data sets. Given that EEG signals are captured across several channels, CNNs are ideal for automatically learning and extracting spatial information from the data. This is especially important since brain activity across the scalp exhibits spatial distribution patterns, and CNNs excel at detecting complicated spatial patterns indicating varying levels of acrophobia reflected in EEG signals. In contrast, ANNs were used in our classification system to supplement CNN capabilities by capturing the temporal dynamics found in EEG data. ANNs, with their fully connected layers and ability to learn complicated correlations within sequential data, excel at handling temporal features of EEG signals, such as changes in brain activity over time. By incorporating ANNs alongside CNNs, we hoped to use the complimentary strengths of both architectures to properly capture the delicate interplay between spatial and temporal variables inside the EEG data, thus boosting the overall accuracy and robustness of our classification model.

Moreover, our decision to utilize well-established CNNs and ANNs aligns with the principle of ensuring reproducibility in scientific studies. These architectures have been extensively studied and validated in various applications, providing a standardized and transparent framework for training, validation, and testing. By employing widely recognized neural network architectures, we enhance the credibility and reliability of our findings, as other researchers can easily replicate our experiments and verify the results using the same methodologies.

#### 2.3.1 Machine learning approaches

Machine learning (ML) algorithms are famous methods for EEG data classification, especially in the field of the current study. Both supervised and unsupervised ML methods have been used in similar studies to interpret the EEG signals. In this study, we try to take advantage of the supervised methods to create a prediction algorithm based on the labeled EEG feature vectors. This algorithm will predict discrete outcomes representing the EEG signal class based on the 4-choice scale, where 0 is for relaxation, 1 for low fear, 2 for medium fear, and 3 for intense fear. Our experimental study will employ the SVC, KNN, RF, Decision Tree, LDA, and Adaboost as ML classifiers to evaluate their effectiveness for accurately classifying the EEG signals into multiple classes. The results of each method will be compared with the other proposed methods used in this experiment.

#### 2.3.2 Artificial Neural Network model

**Figure 2:**
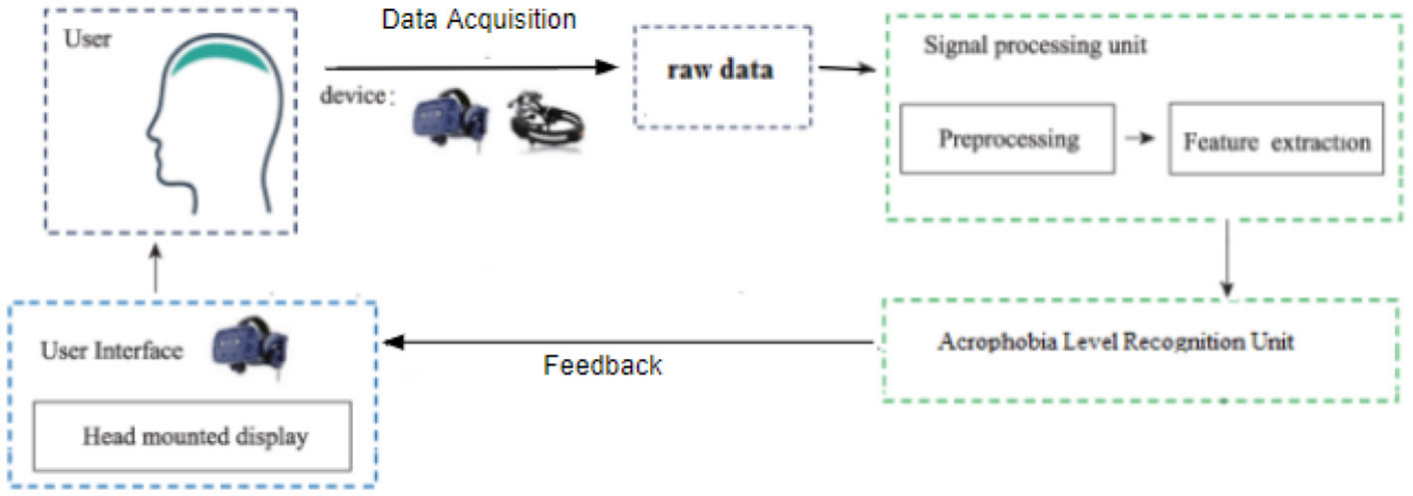
Layout of the proposed model.

The proposed model is a Keras sequential model for multi-class classification. The model consists of three hidden layers of 100 neurons in each layer with a relu activation function. The input shape was 42, which represents the feature vector extracted from the EEG raw data. Four neurons were used in the output layer, with a softmax activation function coresponding to the predicted fear level from 1 to 4. To compile the model, an adam optimizer with a learning rate of 0.01 and sparse categorical cross-entropy loss function were used. The data had been normalized to have 0 means before training and testing. The model was trained on 80% of the total dataset and tested on the remaining 20% during 120 epochs with a batch size of 100 samples. The architecture of the proposed model and hyperparameters are shown in the Figure 2 and Table 3, respectively.

**Table 3:** Hyper-parameters of ANN model.

**No**.	**Hyper-parameter**	**Value**
1	Number of hidden layers	3
2	Number of neurons in each hidden layer	100
3	Activation function	Relu
4	Learning rate	0.01
5	Batch size	100
6	Number of epochs	120
7	Train set	80%
8	Test set	20%
9	Optimizer	Adam
10	Loss function	SPCE

#### 2.3.3 Convolutional Neural Networks

Convolutional Neural Networks (CNNs) are one of the most effective methods for EEG signal classification in the DL field. The motivation behind using this technique for our experiment lies in its ability to capture temporal and spatial patterns, especially with the high dimensional data. Using Pytorch library we create a timeseries CNN model that takes time series data as an input with a window size of 30 samples and 42 features. The model comprises three identical blocks, each consisting of a dropout layer with a dropout rate of 0.2, a Conv1D convolutional layer with a kernel size of 3, and a batch normalization layer with a Relu function for activation. The same padding was used for all blocks to preserve the same dimension of the output as the input. The outcomes of each block represent the input of the following block. The batch normalization layer was introduced to normalize the outputs of each convolutional layer across the mini-batch during training. It facilitates and speeds up the training and prevents overfitting. The dropout layer was used to keep the model from becoming overfit. The convolutional layers 2D tensor was converted to a 1D tensor using a flattened layer. A fully connected layer with a size of (16*42) and an output of four classes that refer to the fear levels is then applied to the outcome after it has been flattened. Finally, the Adam optimizer with a learning rate of 0.001 and the Cross-Entropy loss function were applied. 80% of the total samples were used to train the model, 10% for validation, and 10% for testing. The model architecture and hyperparameters are described in the Figure 2 and Table 4, respectively.

**Table 4:** Hyper-parameters of TimeSeriesCNN model.

**No**.	**Hyper-parameter**	**Value**
1	Number of epochs	100
2	Time series window size	30
3	Time series stride	2
4	Train set	80%
5	Validation set	10%
6	Test set	10%
7	Optimizer	Adam
8	Learning rate	0.001
9	Dropout rate	0.2
10	Batch size	10
11	Loss function	Cross-entropy
12	Number of convolutional layers	3
13	Number of Filters	1
14	Padding	1
15	Stride of conv1D	1

## 3 Results and discussion

For all the models we worked with a dataset of 1940 samples of 42 EEG extracted features. Their output is the estimated fear level from 1 to 4 scale.

### 3.1 Machine learning approaches

Machine learning (ML) approaches, notably supervised algorithms, have a long history of being employed for EEG data classification. This study aligns with the prevailing pattern, aiming to forecast different degrees of terror encountered by participants. The target classes are organized in a systematic manner using a 4-choice scale, which places a high importance on the algorithms' ability to effectively distinguish between these groups.

Based on the obtained data, it is evident that there is a substantial variation in the performances of the algorithms (see Table 5).

**Table 5:** ML classifiers accuracies.

**Classifier**	**Accuracy**
Support vector classifier (SVC)	69%
Decision tree classifier (DT)	53%
Random forest classifier (RF)	75%
K-nearest neighbors classifier (KNN)	86%
Ada boost classifier	50%
Linear discriminant analysis (LDA)	51%

#### 3.1.1 The K-nearest neighbors classifier

The KNN has demonstrated the highest level of accuracy, reaching an astonishing 86% performance. One of the key advantages of the K-nearest neighbors (KNN) algorithm is its non-parametric character, which enables it to effectively capture intricate decision boundaries. The performance of the EEG feature space demonstrates the presence of distinct clusters of data points that correspond to specific classes, and the K-nearest neighbors (KNN) algorithm is capable of effectively distinguishing between these clusters.

#### 3.1.2 The Random Forest Classifier

The RF has commendable performance, with an accuracy rate of 75%. Due to its ensemble structure, Random Forest (RF) demonstrates proficiency in handling high dimensionality and effectively mitigating potential overfitting by employing bagging and random feature selection procedures. The success of the EEG features implies that they may demonstrate specific decision trees that, when combined, contribute to a robust classification capacity.

#### 3.1.3 The Support Vector Classifier

The SVC is a robust method commonly employed for classification tasks, particularly when confronted with data sets including numerous dimensions. It has been observed to possess a satisfactory accuracy rate of 69%, rendering it a dependable approach for various classification challenges. The objective is to identify the optimal hyperplane that effectively separates distinct classes. The satisfactory performance can be due to the inherent characteristics of EEG data, which allow for the successful division of different fear levels using either a linear or non-linear hyperplane (with an appropriate kernel).

#### 3.1.4 Linear Discriminant Analysis

The LDA exhibits a modest performance with an accuracy of 51% for a 4-class problem, indicating that its predictive capability is just marginally better than random chance. The objective of this approach is to optimize the separation between the means of distinct classes while simultaneously minimizing the dispersion (variance) within each class. The average performance of the model may suggest the presence of a substantial intersection among the classes or non-linear boundaries within the dataset, which the Linear Discriminant Analysis (LDA) method is unable to accurately represent.

#### 3.1.5 Decision Tree Classifier

The performance of the Decision Tree Classifier is ~53%, which is close to the baseline. Decision Trees partition the data by utilizing specific thresholds associated with features. The inadequate performance shown implies that employing basic threshold-based divisions on individual attributes alone is insufficient for effectively differentiating between the classes.

#### 3.1.6 The AdaBoost classifier

With an accuracy rate of 50%, exhibits sub-optimal performance on the given data set. The under-performance of the ensemble method can be attributed to the inherent limitations of the individual weak classifiers, typically decision trees, in effectively classifying the EEG data. The application of boosting techniques does not yield substantial improvements in accuracy.

In conclusion, it can be observed that KNN and RF had notable performances, but AdaBoost, DT, and LDA demonstrated comparatively subpar results in the given job. The diverse levels of achievement observed among algorithms provide insight into the intrinsic intricacies and subtleties of the electroencephalogram (EEG) data. Moreover, this observation implies the existence of intricate and non-linear patterns within the EEG data, which may be more effectively captured by some algorithms compared to others. The subsequent steps may encompass the optimisation of hyper-parameters for the under-performing algorithms or the incorporation of domain-specific expertise to enhance the feature set or the selection of methods.

### 3.2 Artificial Neural Network

**Figure 3:**
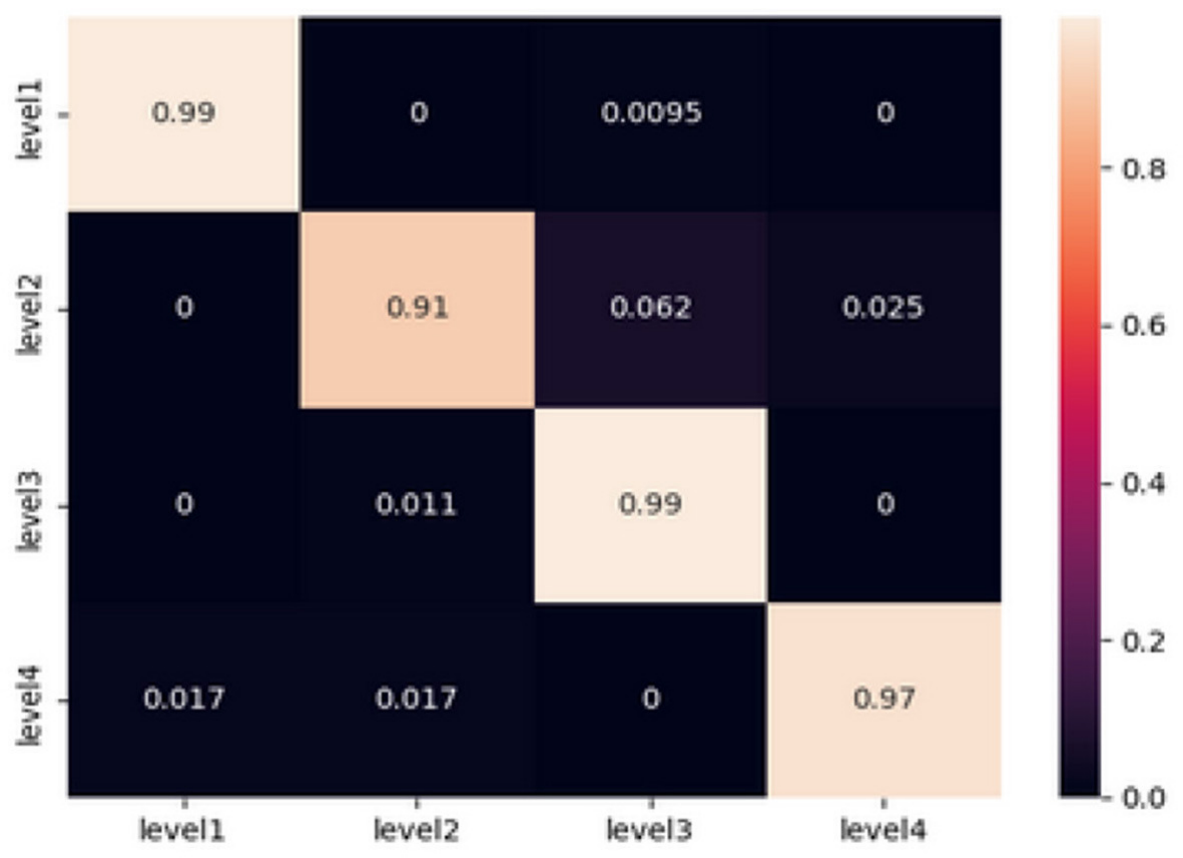
Confusion matrix obtained from testing the ANN model.

Artificial neural networks, particularly feed-forward topologies, have gained recognition for their capacity to effectively mimic any continuous function given a sufficient number of neurons. These characteristics render them highly suitable candidates for a wide range of classification tasks. The inherent ability of individuals to effectively handle non-linearities is crucial, given the complex characteristics exhibited by EEG signals. The sequential Keras model that we have selected consists of three hidden layers. In this architecture, every layer contains 100 neurons and employs the rectified linear unit (ReLU) activation function. The selection of ReLU is attributed to its advantageous property of having a non-vanishing gradient. The artificial neural network (ANN) model being investigated was developed and evaluated using the same dataset that was used to train the machine learning (ML) classifiers. The dataset consists of 1940 samples, where each entry contains 42 features taken from EEG signals. In terms of training, the model exhibited a 96% accuracy rate along with a loss value of 0.32. During the testing phase, the system demonstrated a noteworthy accuracy rate of 97%, resulting in a loss of 0.24. The correctness of the testing is determined by calculating the values from the confusion matrix, which is illustrated in the Figure 3.

In order to conduct a more comprehensive analysis, the confusion matrix provides a detailed examination of the accurate and inaccurate categorizations produced by the artificial neural network (ANN).

At Level 1 (relaxation), the artificial neural network (ANN) model demonstrated its exceptional performance by achieving a 99% accuracy rate. A negligible misclassification rate of 0.95% was recorded specifically for level 3, whereas no misclassifications were observed for levels 2 and 4.

Level 2 (low fear): The findings indicate a modest amount of challenge, as indicated by misclassifications of 6.2 and 2.5% toward level 3 and level 4, respectively, with an overall accuracy rate of 91%.

At a forecast accuracy of 99%, the level of precision is commendable. A marginal misclassification rate of 1.1% was observed specifically for level 2, while no misclassifications were detected for levels 1 and 4.

The classification model achieved a high accuracy rate of 97% in predicting Level 4 (Intense Fear). However, there were minor misclassifications of 1.7% each, which were seen in levels 1 and 2. These misclassifications serve to emphasize the model's effectiveness in accurately detecting extreme emotional states.

The comprehensive analysis highlights the model's ability to accurately differentiate between different levels of fear intensity based on EEG signals. The presence of minor misclassifications, particularly in cases when fear intensities are closely related, highlights the intricate and nuanced characteristics of these emotional thresholds. The results of this study raise inquiries on the underlying parallels in EEG patterns observed between successive levels of terror intensity. This observation suggests the possibility of further improving the model or incorporating more variables to accurately capture these subtle distinctions.

In summary, the sequential artificial neural network (ANN) model, with its layered architecture, has demonstrated notable proficiency in classifying EEG signals, highlighting its potential as a reliable tool for detecting emotions based on EEG data.

### 3.3 Convolutional Neural Network

The suggested TimeSeries CNN model was subjected to validation and testing using a dataset of consistent dimensions, specifically 1,940 × 42. The model received time series data as its input. From this data, a total of 913 tensors were retrieved using a window size of 30 and a stride of 2. The main aim of the model was to assess the level of fear, which was classified into four discrete categories, ranging from 1 to 4.

**Figure 4:**
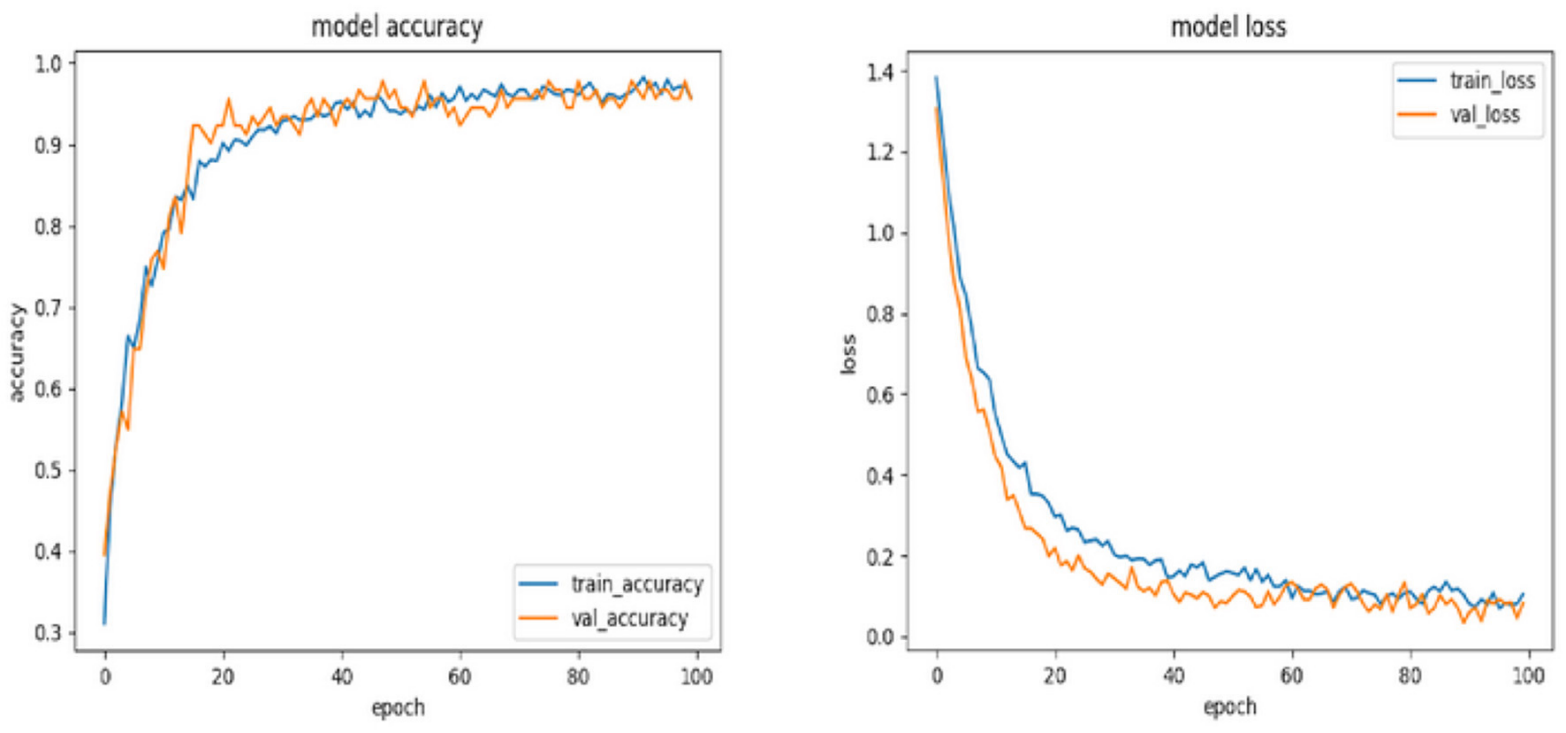
Plots for training and validation accuracies and losses of the time series CNN model.

**Figure 5:**
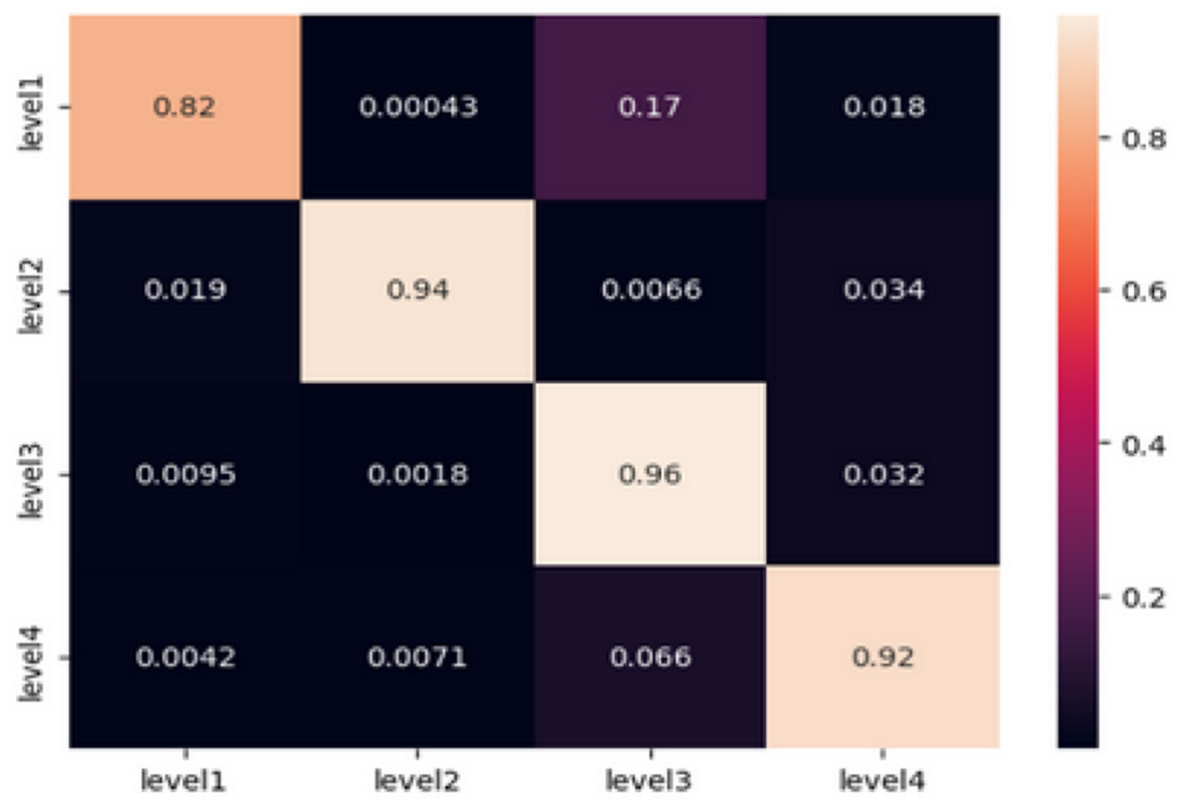
Confusion matrix obtained from testing the TimeSeriesCNN model.

The accuracy and loss metrics of the model have demonstrated noteworthy performance across its whole lifespan. Specifically, the model achieved an accuracy score of 96% during the training phase, 98% during the validation phase, and reached its highest accuracy of 99% during the testing phase (see Figures 4, 5). The measures demonstrate the model's strong capacity to generalize across several data segments, suggesting that overfitting is not present. The claim is reinforced by the consistent loss values that were recorded, namely 0.1 during the training phase, 0.03 in the validation phase, and 0.05 during the testing phase. The observed trend of enhanced accuracy and reduced loss throughout the transition from training to testing indicates that the model has been effectively optimized and possesses the capability to effectively process the complex EEG time series data.

The classification of the “Level 1” category demonstrates a noteworthy strength, as indicated by an accuracy rate of 0.82. However, it has been noticed that the model frequently confuses “Level 1” with “Level 3,” resulting in an error rate of ~17%. The model demonstrates excellent performance in predicting “Level 2” with a high accuracy of 0.94, with only minimal misclassifications in relation to the other levels. The model's prediction accuracy at Level 3 is noteworthy, with a score of 0.96. However, there are minor discrepancies seen, particularly toward Level 4. In the case of “Level 4,” the model demonstrates a commendable accuracy rate of 0.92. However, it is worth noting that there is a noticeable misclassification rate of 6.6% toward the “Level 3” category. The provided confusion matrix offers valuable insights into the specific categorization issues encountered by the model. Although the accuracy metrics as a whole are commendable, there are certain repeating patterns of misclassification, such as the confusion between “Level 1” and “Level 3.” This phenomenon may arise due to the existence of shared electroencephalogram (EEG) characteristics at these levels or specific complexities that the model may not have fully comprehended.

In conclusion, the TimeSeries CNN has exhibited exceptional proficiency in categorizing EEG time series data into different fear categories, owing to its customized architecture and optimized hyperparameters. The accuracy and loss graphs supplied, along with the analysis of the confusion matrix, validate the reliability of the model in this particular field. In order to optimize future undertakings, it is advisable to concentrate on the enhancement of particular domains of misclassification. Potential strategies encompass a spectrum of methods, including the incorporation of domain-specific information, augmentation of the training dataset, and adjustment of the architecture and hyperparameters.

### 3.4 Comparative study

In comparison to previous works in the field of acrophobia treatment using EEG data analysis and machine learning techniques, our proposed TimeSeries CNN model and artificial neural network (ANN) model demonstrate notable strengths and areas for improvement.

Compared to Study Balan et al. (2019), which utilizes neural networks for fear estimation in a VR game, our TimeSeries CNN model achieves higher accuracy rates across all fear levels during both training and testing phases. Specifically, our model achieves an accuracy score of 99% during the testing phase, surpassing the accuracy rates reported in Study Balan et al. (2019). However, similar to Study Balan et al. (2019), our model also exhibits confusion between “Level 1” and “Level 3,” suggesting a potential area for further refinement in fear level classification.

Similarly, in comparison to Study Bălan et al. (2020), which investigates machine learning classifiers for fear level estimation in a VR system, our TimeSeries CNN model demonstrates superior performance in predicting fear levels, particularly achieving higher accuracy rates for “Level 1” and “Level 3.” Additionally, our model showcases consistent accuracy improvement from training to testing phases, indicating effective optimization and generalization capabilities.

Compared to the ANN model evaluated in our work, Study Apicella et al. (2023) proposes an EEG-based classification system for fear of heights using VR scenarios. While both studies achieve high accuracy rates for fear level prediction, our ANN model demonstrates exceptional accuracy of 99% for “Level 1” and “Level 3,” highlighting its robustness in detecting relaxation and moderate fear states. However, similar to Study Apicella et al. (2023), our model exhibits misclassifications between adjacent fear levels, indicating potential challenges in distinguishing between subtle variations in fear intensity.

Compared to Study Tychkov et al. (2023), which explores the use of spectral analysis of EEG power for assessing psychoemotional states in acrophobia using VR technology, our proposed TimeSeries CNN and ANN models offer complementary approaches for fear level classification. While Study Tychkov et al. (2023) focuses on spectral analysis of EEG power to identify markers for anxious-phobic disorders, our models directly classify fear levels based on EEG signals. Our models demonstrate superior accuracy rates for fear level prediction, particularly achieving high accuracy for “Level 1” and “Level 3.” However, similar to Study Tychkov et al. (2023), our models may encounter challenges in distinguishing between closely related emotional states, as evidenced by misclassifications between adjacent fear levels.

Overall, our proposed TimeSeries CNN and ANN models exhibit promising performance in fear level classification, showcasing strengths in accurately predicting different fear states. However, further investigation is warranted to address specific misclassification patterns observed across fear levels and enhance the models' ability to differentiate between closely related emotional states.

### 3.5 Exploratory data analysis and statistical testing of EEG feature sets

In the present study, we conducted an examination of exploratory data analysis (EDA) and statistical testing on several sets of Electroencephalography (EEG) features, as documented in the works of Maghsoudi and Shalbaf (2022) and Staji et al. (2022). Electroencephalography (EEG) is a non-invasive technique used to record the electrical activity of the brain. In the context of our research, we observed distinct characteristics in the form of mean frequencies, specifically Alpha, Beta, and Gamma, as shown in the studies by Rochais et al. (2018) and Lin and Fang (2021). Out of the three feature extraction methods utilized in our study, the approach that utilized Power Spectral Density (PSD) through STFT method emerged as the most prominent. This method yielded 42 unique features, which corresponded to the 14-channel EEG headset employed in our research. Notably, these features exhibited superior classification outcomes. However, it is important to note that we solely focused on optimizing the analysis by utilizing the mean of each wave. In this study, we conducted an analysis of descriptive statistics to compute the measures of central tendency (means) and variability (standard deviations). The findings are shown in Table 6. The study utilized Analysis of Variance (ANOVA) using different python libraries (MNE, Pingouin, Pandas, Scipy) and interpreted manually to assess the presence of statistically significant differences across the Alpha, Beta, and Gamma frequency bands across four stimuli. The corresponding averages for each frequency band and stimulus are presented in Table 7. The null hypothesis in ANOVA testing posits that there are no significant differences between the groups being compared, and any observed deviations are exclusively attributable to random sampling variability. We performed an exhaustive power analysis utilizing G*Power to address concerns pertaining to the adequacy of our sample size in detecting significant and subtle effects in EEG features across various levels of acrophobia. The power analysis shows that our sample size is relevant enough to obtain a statistical power <0.8.

Alpha mean: The analysis of variance (ANOVA) test conducted on the Alpha mean yielded an *F*-value of 2.002, accompanied by a *P*-value of 0.112. Despite the fact that the observed *P*-value does not fall below the commonly accepted alpha level of 0.05, it does indicate a discernible pattern indicating potential variations in the mean Alpha values across various levels of acrophobia. Nevertheless, it is not possible to definitively dismiss the null hypothesis, suggesting that there is no statistically significant variation in the average Alpha frequency responses across the four acrophobia stimuli.
Beta mean: The *F*-value obtained for the ANOVA test on the Beta mean was 7.119, indicating a statistically significant result. The associated *P*-value was found to be very significant (*P* < 0.01), about 9.357e-05. Based on the obtained results, our findings indicate that we fall significantly below the predetermined alpha level of 0.05, hence providing sufficient evidence to reject the null hypothesis. The results of this study demonstrate statistically significant variances in the average Beta frequency responses across the different stimuli associated with acrophobia. These findings reflect noteworthy disparities in the levels of active, busy, or nervous thinking and concentration exhibited by the participants.
Gamma mean: The ANOVA test conducted on the Gamma mean resulted in an *F*-value of 15.410, indicating a very significant relationship. The associated *P*-value was found to be <0.01, specifically roughly 6.578e-10. Consequently, the null hypothesis about the mean Gamma frequency responses for diverse acrophobia stimuli is rejected, indicating the presence of statistically significant variations. These findings indicate notable disparities in higher-order cognitive processing and information processing among the stimuli.

**Table 6:** Descriptive statistics of EEG features for different acrophobia levels.

	**Alpha mean**	**Beta mean**	**Gamma mean**
**Level 1 (lowest acrophobia level)**			
Count	498	498	498
Mean	2.76	0.69	–6.06
Std	3.01	3.09	3.83
Min	–3.46	–5.69	–13.90
Max	18.15	13.59	12.86
**Level 2 (mild acrophobia level)**			
Count	417	417	417
Mean	2.79	1.00	–5.74
Std	3.84	4.18	4.430
Min	–4.67	–5.24	–12.84
Max	18.13	13.68	7.77
**Level 3 (moderate acrophobia level)**			
Count	462	462	462
Mean	3.19	0.84	–6.07
Std	2.63	2.62	2.90
Min	–2.60	–3.64	–11.22
Max	32.79	21.50	9.36
**Level 4 (highest acrophobia level)**			
Count	563	563	563
Mean	3.04	1.59	–4.66
Std	3.16	3.64	4.29
Min	–4.37	–5.85	–11.47
Max	19.56	13.52	8.88

**Table 7:** The obtained results of the statistical test.

	**Alpha mean**	**Beta mean**	**Gamma mean**
*F*-value	2.00	7.11	15.40
*P*-value	0.11	<0.01	<0.01

In the subsequent research, we explore the statistical interpretations of electroencephalography (EEG) data in relation to different levels of acrophobia. Significant differences in the mean values across diverse stimuli were seen through the ANOVA tests run on the Alpha, Beta, and Gamma frequency bands. Nevertheless, it is crucial to acknowledge that ANOVA in itself does not precisely identify the specific distinctions between every set of stimuli. In order to effectively discern the specific differences across stimulus levels, it is important to conduct further *post-hoc* testing. The inclusion of these supplementary analyses is crucial not only for comprehending the subtle distinctions among each level of acrophobia but also for effectively managing the heightened risk of Type I errors that frequently arise when doing numerous statistical comparisons. By employing a careful methodology, our objective is to offer a comprehensive comprehension of the electroencephalogram (EEG) data, therefore illuminating the subtle neurophysiological dynamics that form the basis of acrophobia.

Level 01: At the most basic level of acrophobia, the mean Alpha wave activity is recorded as 2.76, with a standard deviation of 3.01. This suggests a significant range of variability in Alpha wave activity across the participants. The observed range of relaxation responses, spanning from –3.46 to 18.15, encompasses a wide spectrum. The Beta mean, which is calculated to be 0.69, exhibits a notable standard deviation of 3.09, indicating a wide range of cognitive involvement levels among the individuals. The mean value of the negative Gamma activity, –6.06, accompanied with a standard deviation of 3.83, signifies a decrease in Gamma activity. This decrease is frequently associated with cognitive activities.
Level 02: At this particular level, the mean value of Alpha exhibits a modest increase, reaching 2.79. Additionally, the standard deviation of Alpha extends to 3.84, indicating a higher degree of variability in the relaxation responses observed among the subjects. The Beta mean exhibits an increase to 1.00, accompanied by a higher standard deviation of 4.18. This suggests the presence of heightened alertness or cognitive stress among certain individuals. The Gamma score, which has a mean of –5.74 and a standard deviation of 4.43, indicates a cognitive reaction that is both more uniform and intense.
Level 03: The Alpha mean demonstrates a notable increase to 3.19, accompanied by a decreased standard deviation of 2.63. This suggests a heightened and more consistent relaxation response. The Beta mean exhibits a little decline to 0.84, accompanied by a standard deviation of 2.62. This observation implies a tendency toward a more homogeneous degree of awareness. The Gamma mean, which is seen to be –6.07, exhibits a resemblance to Level 1, accompanied with a standard deviation of 2.90. This finding suggests a consistent pattern in cognitive processing among the participants.
Level 04: In the most extreme case of acrophobia, the mean value of Alpha experiences a modest decline to 3.04ease to 3.16. This observation implies a greater degree of variability in stress responses. The Beta mean exhibits a notable increase to 1.59, accompanied by a substantial standard deviation of 3.64, indicating a significant level of variability in cognitive stress. The Gamma mean, which is seen to be –4.66 with a standard deviation of 4.29, suggests a range of cognitive processing abilities that may be diverse and potentially enhanced.

Correlation studies were conducted to enhance our comprehension of the EEG feature sets and their interrelationships across various levels of acrophobia. Correlation matrices provide a measurable indication of the extent to which two variables are associated. The heatmaps presented below depict the correlation matrices corresponding to different levels of acrophobia. These heatmaps offer a graphical representation of the magnitude and direction of the associations between the mean frequency bands of Alpha, Beta, and Gamma. Matrices play a crucial role in the identification of patterns that may not be readily discernible alone through the use of ANOVA.

Upon analyzing the matrices, it becomes evident that the correlation coefficients exhibit a spectrum of values ranging from modest to high. This signifies the presence of diverse levels of linear relationship between the distinct EEG frequency bands.

Level 01: The matrix demonstrates a robust positive correlation (*r* = 0.9) between the means of Beta and Gamma activity, indicating that a rise in Beta activity is associated with a corresponding increase in Gamma activity within the lowest level of acrophobia. The variables Alpha and Beta, as well as Alpha and Gamma, have a statistically significant positive link, with correlation coefficients of 0.74 and 0.56, respectively.
Level 02: In this observation, a robust positive correlation is shown between the means of Beta and Gamma (0.95), surpassing the strength observed at Level 1. The Alpha mean exhibits a significant association with both the Beta and Gamma averages, with correlation coefficients of 0.86 and 0.79, respectively. This suggests a higher degree of synchronized activity between these frequency bands in individuals with acrophobia at this particular level.
Level 03: The correlations exhibit a little decline while maintaining a significant strength, as evidenced by the high correlation coefficient (0.89) observed between the means of Beta and Gamma. The Alpha mean demonstrates a robust positive association with both the Beta mean (0.73) and the Gamma mean (0.62), which aligns with the observed trend in prior levels.
Level 04: At the acrophobia level characterized as the greatest, there exists a strong correlation (0.94) between the means of Beta and Gamma, which is comparable to the correlation observed at Level 2. The Alpha mean has a connection of moderate to strong magnitude with Beta (0.7) and a correlation of moderate magnitude with Gamma (0.57).

The observed results suggest a continuous association between the EEG frequency bands and various levels of acrophobia. The Beta and Gamma frequency bands, which are frequently linked to cognitive processing and engagement, consistently exhibit a robust positive association. This observation may indicate the cognitive and emotional challenges associated with the processing of cues that induce acrophobia. The results indicates that there is a positive correlation between the severity of acrophobia and the degrees of Beta and Gamma activity. This correlation suggests that persons with greater levels of acrophobia have increased cognitive arousal and engagement.

### 3.6 Cronbach's alpha

In psychometrics, Cronbach's alpha stands as a fundamental measure of internal consistency reliability, pivotal in gauging the reliability of scales or sets of items within various psychological constructs (Tavakol and Dennick, 2011). It serves to evaluate the degree to which items within a scale or measure exhibit consistent patterns of response, essentially quantifying the extent to which items correlate with each other. By doing so, Cronbach's alpha provides researchers with valuable insights into the reliability and consistency of the measurements obtained from multiple items or channels (Vaske et al., 2017).

The formula to calculate Cronbach's alpha is as follows:


(1) $$\begin{array}{l} \alpha =\frac{n-1}{n}\left(1-\frac{\sigma_{total}^{2}}{\sum \sigma_{item}^{2}}\right) \end{array}$$

In Equation 1, α represents Cronbach's alpha, *n* denotes the number of items or channels, $\sigma_{item}^{2}$ signifies the variance of individual items, and $\sigma_{total}^{2}$ represents the total variance.

In the context of our study, which explores the classification of EEG signals from individuals with varying levels of acrophobia in virtual reality, the utilization of Cronbach's alpha is instrumental. By evaluating the internal consistency and reliability of the EEG data, we aim to ascertain the robustness of our measurements and the consistency of neural activity patterns across different channels.

After combining data from four distinct CSV files, each containing EEG recordings corresponding to different acrophobia levels, we proceeded to calculate Cronbach's alpha. The resulting value, ~0.886, with a 95% confidence interval ranging from 0.881 to 0.891, signifies a high level of internal consistency among the EEG channels measured in our study. This outcome suggests that the EEG channels reliably capture the underlying neural activity associated with varying levels of acrophobia in our participant cohort.

Such findings underscore the validity and reliability of our EEG data, substantiating its suitability for further analysis and interpretation. The robust internal consistency revealed by Cronbach's alpha reinforces the confidence in the integrity of our measurements, thus bolstering the credibility of our study outcomes.

### 3.7 EEG analysis

The findings derived from the analysis of descriptive statistics, analysis of variance (ANOVA), and correlation matrices offer a comprehensive understanding of the neurophysiological reactions of acrophobia, as assessed using electroencephalography (EEG). The descriptive statistics reveal a discernible pattern in the data, wherein the average frequencies of Alpha, Beta, and Gamma waves exhibit a consistent tendency across various levels of acrophobia. An evident correlation exists between the escalating levels of acrophobia and the average Alpha and Beta frequencies, suggesting a potential augmentation in alertness and cognitive involvement in reaction to stimuli that induce fear.

The findings of the analysis of variance (ANOVA) highlight this fact, indicating notable disparities in the levels of Beta and Gamma wave activities among individuals with different degrees of acrophobia. However, there is no statistically significant variation observed in Alpha wave patterns. The obtained *F*-value of 15.409 for Gamma wave activity, along with the very significant *P*-value (*P* < 1e-9), provides compelling evidence that Gamma wave activity is highly responsive to the degree of acrophobia. This finding perhaps indicates that the heightened cognitive requirements associated with fear processing contribute to the observed sensitivity. The Beta waves, which exhibit notable variations across different levels of acrophobia, may potentially indicate an individual's increased vigilance and anxiety when exposed to stimuli related to acrophobia.

The correlation matrices illustrate the associations between various brainwave frequencies, visually representing the relationships. These matrices consistently demonstrate a robust positive correlation between Beta and Gamma waves. This observation suggests that as individuals are exposed to more heightened acrophobic stimuli, there is a corresponding elevation in both cognitive processing, as indicated by Gamma activity, and emotional stress or anxiety, as indicated by Beta activity. The observed positive correlations indicate that the brainwave activities under investigation may not be independent entities, but rather components of a coordinated reaction to acrophobia.

The findings indicate that there are moderate correlations between Alpha wave activity and the other two frequencies. These connections show that as acrophobia severity grows, Alpha activity also increases. However, it appears that the relationship between Alpha activity and cognitive and emotional stress responses may not be as strong as the relationship between Beta and Gamma waves and these reactions. This phenomenon may be attributed to the conventional correlation between Alpha waves and states of relaxation, which may have a less direct impact on the immediate stress reaction but can still be influenced by the individual's overall emotional and cognitive condition.

The integration of these findings, along with the demonstrated high accuracy of the Convolutional Neural Network (CNN) and Artificial Neural Network (ANN) models in identifying acrophobia levels, presents a persuasive rationale for utilizing EEG data in comprehending and perhaps diagnosing acrophobia. The robust performance exhibited by these models serves to confirm the EEG data's credibility as a dependable information source concerning acrophobia. Furthermore, it highlights the capacity of machine learning methodologies to discern significant patterns from intricate neurophysiological data.

In summary, the findings of this study indicate that electroencephalography (EEG) has the potential to be a valuable instrument for evaluating acrophobia. The examination of distinct brainwave frequencies provides valuable information regarding the cognitive and emotional aspects of this particular fear. The results of the study also support the notion of adopting a holistic perspective in comprehending acrophobia, wherein cognitive processes and emotional reactions are recognized as interrelated components of the acrophobic phenomenon.

### 3.8 Brain correlation

Brain correlation refers to the statistical relationship between different brain regions or neural activities, typically measured using neuroimaging techniques such as electroencephalography (EEG) or functional magnetic resonance imaging (fMRI). In the context of this study, brain correlation specifically refers to the degree of association or synchronization between the activities of different frequency bands, such as Alpha, Beta, and Gamma, within specific regions of the brain.

**Figure 6:**
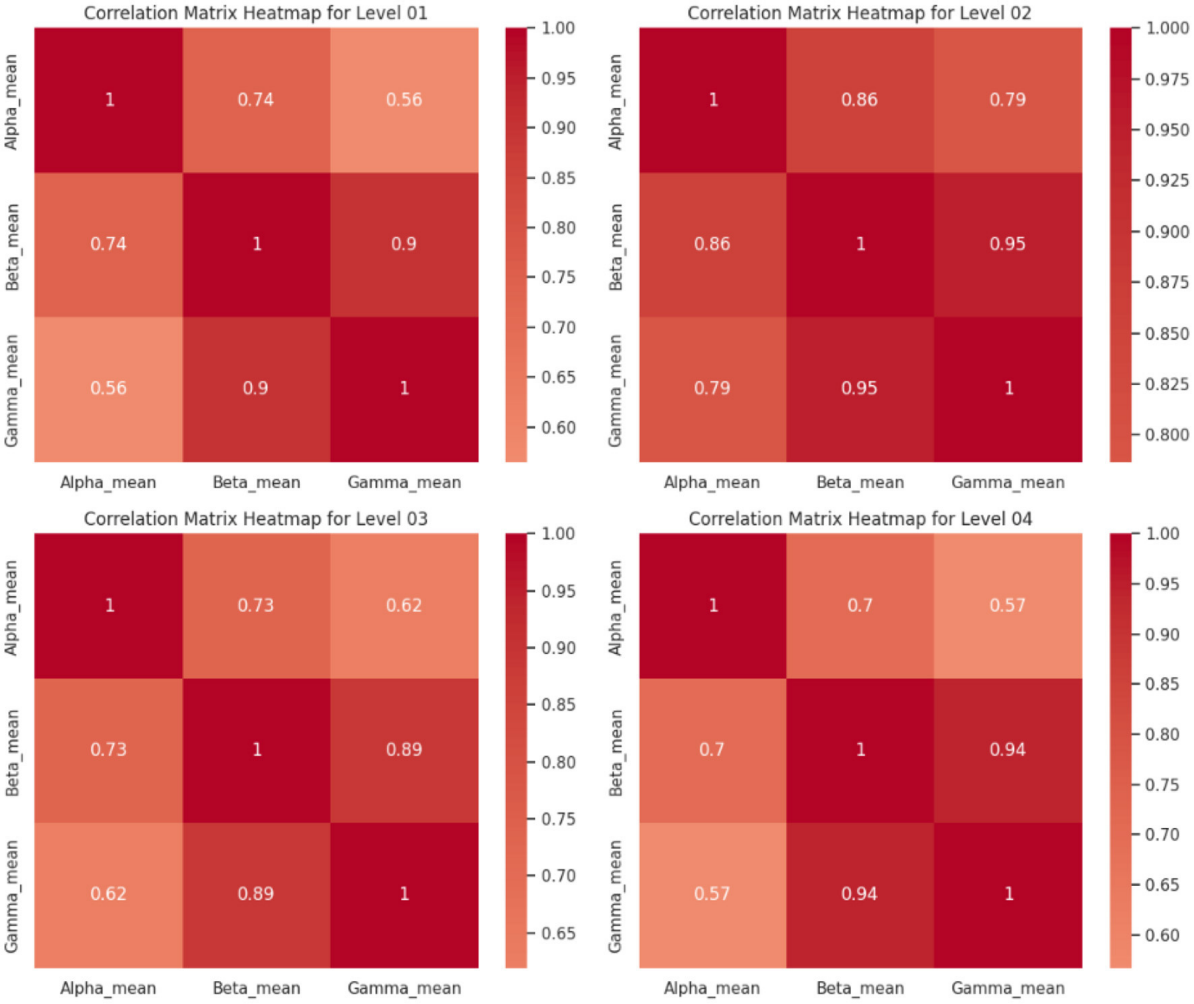
Correlation matrices.

The results presented in Figure 6 demonstrate robust positive correlations between the means of Beta and Gamma activities across different levels of acrophobia. These correlations indicate that changes in Beta activity are consistently associated with corresponding changes in Gamma activity within each level of acrophobia. Additionally, significant positive correlations were observed between Alpha and Beta, as well as Alpha and Gamma activities, suggesting synchronized activity across these frequency bands.

Furthermore, the strength of these correlations varied across acrophobia levels, with higher levels of acrophobia exhibiting slightly weaker but still significant correlations. These findings suggest that the degree of synchronization between different brain regions or frequency bands may vary depending on the severity of acrophobia, highlighting the complex interplay between neural activities in response to fear-inducing stimuli.

Overall, these results underscore the importance of understanding brain correlations in the context of acrophobia, as they provide valuable insights into the underlying neural mechanisms associated with fear processing and potentially inform the development of targeted interventions or treatments for acrophobia.

### 3.9 Limitations

While our study presents promising results in fear level classification using TimeSeries CNN and ANN models, several limitations should be acknowledged. Firstly, the dataset's relatively small sample size may restrict the generalizability of our findings to broader populations, necessitating validation on larger and more diverse datasets, since our dataset included only Adults. Additionally, our models rely solely on EEG signals, neglecting the potential benefits of incorporating other modalities such as Galvanic Skin Response (GSR) or Heart Rate (HR), which could enhance classification accuracy. Furthermore, individual differences in EEG patterns and subjective experiences of fear may introduce variability in classification performance, highlighting the need to control for factors like age, gender, and previous exposure to acrophobic stimuli. Despite efforts to mitigate overfitting through validation techniques, the risk of overfitting to the training data remains, underscoring the importance of further validation on independent datasets. Lastly, while our models achieve high accuracy rates, their interpretability remains limited, necessitating additional efforts to understand the underlying neural mechanisms contributing to fear classification. Addressing these limitations through larger-scale studies, multimodal data integration, consideration of individual differences, enhanced interpretability, and rigorous validation can strengthen the reliability and applicability of our fear level classification models in real-world scenarios.

## 4 Conclusion

The present work aimed to do an exploratory investigation into the classification of electroencephalogram (EEG) signals across a range of acrophobia levels. By utilizing a combination of conventional machine learning techniques and sophisticated deep learning approaches, we have conducted a thorough examination of electroencephalogram (EEG) data in order to get comprehensive insights into the intricate neurophysiological aspects of acrophobia. The analytical process undertaken in our study encompassed the utilization of spectral analysis techniques to examine brainwave data, doing correlation assessments across different regions of the brain, and extracting relevant features. These methodologies were employed in order to decipher the story sent by electroencephalogram (EEG) signals pertaining to acrophobic responses.

The utilization of Time Series Convolutional Neural Networks (CNN) and Artificial Neural Networks (ANN) within this framework produced remarkable outcomes, demonstrating a notable testing accuracy of over 97%. The precision of our results, particularly demonstrated in the confusion matrices, highlights the proficiency of these models in distinguishing the complex EEG patterns linked to different levels of acrophobia. This serves as evidence of the computational models' capability to effectively handle the intricate and diverse nature of EEG signals.

However, it is important to note that every scientific endeavor possesses its own boundaries of improvement and advancement. The presence of certain misclassification patterns and the varying precision levels seen among machine learning algorithms suggest potential areas for improvement. Additional optimisation could be pursued by enhancing feature engineering, augmenting the data, and incorporating domain-specific knowledge.

Notwithstanding the potential for additional investigation, the advancements achieved in this study serve as a symbol of forward movement. Our research not only provides a detailed quantitative analysis but also lays the foundation for a more profound integration of artificial intelligence with the fields of neurology and psychology. We are currently at the threshold of a period in which the convergence of cutting-edge technology and cognitive research holds the potential to enhance our comprehension of the intricate workings of the human brain. With the ongoing development of artificial intelligence and the increasing availability of data, it is expected that increasingly advanced techniques will be developed for the classification of EEG data. Undoubtedly, this development will advance the confluence of neurology, emotion, and technology, hence augmenting our ability to comprehensively analyse and decipher the complex interplay of human brain and emotional phenomena.

## Data availability statement

The raw data supporting the conclusions of this article will be made available by the authors, without undue reservation.

## Ethics statement

The study was conducted in accordance with the Declaration of Helsinki, and approved by the internal ethics committee at the University of Biskra in Algeria, as well as by the International Trans-disciplinary Ethics Committee for Human-related research (protocol n. 2023021101 approved on March 9 2023). The studies were conducted in accordance with the local legislation and institutional requirements. The participants provided their written informed consent to participate in this study.

## Author contributions

SR: Conceptualization, Data curation, Investigation, Methodology, Writing – original draft. IT: Conceptualization, Data curation, Investigation, Methodology, Software, Writing – original draft. AT: Formal analysis, Validation, Writing – original draft. DC: Resources, Visualization, Writing – original draft. AN: Resources, Validation, Writing – original draft. JS: Formal analysis, Funding acquisition, Supervision, Validation, Writing – review & editing. CN: Conceptualization, Formal analysis, Funding acquisition, Project administration, Supervision, Validation, Writing – original draft.
